# Bis­(methane­sulfonato-κ*O*)(5,10,15,20-tetra­phenyl­porphyrinato-κ^4^ *N*,*N*′,*N*′′,*N*′′′)tin(IV) chloro­form tris­olvate

**DOI:** 10.1107/S1600536812015875

**Published:** 2012-04-18

**Authors:** Atul P. Singh, Hee-Joon Kim

**Affiliations:** a Department of Applied Chemistry, Kumoh National Institute of Technology, 1 Yangho-dong, Gumi 730-701, Republic of Korea

## Abstract

In the crystal structure of the title compound, [Sn(C_44_H_28_N_4_)(CH_3_O_3_S)_2_]·3CHCl_3_, the Sn^IV^ ion is located on an inversion center and is octa­hedrally coordinated. The porphyrin N atoms occupy the equatorial positions while the axial positions are occupied by the O atoms of the methane­sulfonate anions. The phenyl rings make dihedral angles of 77.02 (13) and 87.89 (14)° with the porphyrin ring. Of the three solvent chloro­form mol­ecules, one is disordered over a twofold rotation axis. In the crystal a three-dimensional assembly is accomplished *via* C—H⋯O hydrogen bonds between the H atoms of the phenyl groups in the porphyrin ring and the O atoms of the methane­sulfonate ligands.

## Related literature

For general background to tin(IV) porphyrin chemistry, see: Arnold & Blok (2004 ▶). For the preparation of related tin porphyrins, see: Kim *et al.* (2004 ▶, 2005 ▶, 2007 ▶, 2009 ▶). For related structures, see: Liu *et al.* (1996 ▶); Smith *et al.* (1991 ▶).
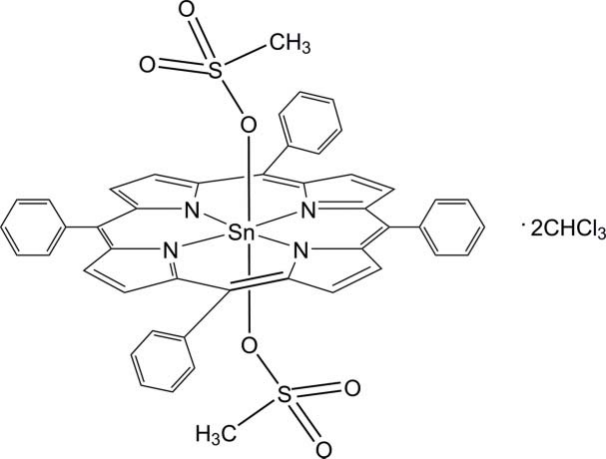


## Experimental

### 

#### Crystal data

[Sn(C_44_H_28_N_4_)(CH_3_O_3_S)_2_]·3CHCl_3_
*M* *_r_* = 1279.69
Monoclinic, 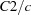

*a* = 25.379 (2) Å
*b* = 11.6269 (9) Å
*c* = 20.860 (3) Å
β = 120.934 (1)°
*V* = 5279.9 (9) Å^3^
*Z* = 4
Mo *K*α radiation
μ = 1.07 mm^−1^
*T* = 150 K
0.26 × 0.19 × 0.16 mm

#### Data collection

Bruker APEXII CCD diffractometer
Absorption correction: multi-scan (*SADABS*; Bruker, 2009 ▶) *T*_min_ = 0.765, *T*_max_ = 0.848
22613 measured reflections
5192 independent reflections
4525 reflections with *I* > 2σ(*I*)
*R*_int_ = 0.035

#### Refinement

*R*[*F* ^2^ > 2σ(*F* ^2^)] = 0.033
*wR*(*F* ^2^) = 0.088
*S* = 1.04
5192 reflections
331 parameters
H-atom parameters constrained
Δρ_max_ = 0.91 e Å^−3^
Δρ_min_ = −0.86 e Å^−3^

### 

Data collection: *APEX2* (Bruker, 2009 ▶); cell refinement: *SAINT* (Bruker, 2009 ▶); data reduction: *SAINT*; program(s) used to solve structure: *SHELXS97* (Sheldrick, 2008 ▶); program(s) used to refine structure: *SHELXL97* (Sheldrick, 2008 ▶); molecular graphics: *SHELXTL* (Sheldrick, 2008 ▶); software used to prepare material for publication: *SHELXTL*.

## Supplementary Material

Crystal structure: contains datablock(s) I, global. DOI: 10.1107/S1600536812015875/wm2606sup1.cif

Structure factors: contains datablock(s) I. DOI: 10.1107/S1600536812015875/wm2606Isup2.hkl

Additional supplementary materials:  crystallographic information; 3D view; checkCIF report

## Figures and Tables

**Table 1:** Hydrogen-bond geometry (Å, °)

*D*—H⋯*A*	*D*—H	H⋯*A*	*D*⋯*A*	*D*—H⋯*A*
C8—H8*A*⋯O2^i^	0.95	2.55	3.280 (4)	134
C24—H24*A*⋯O2^ii^	1.00	2.35	3.191 (4)	141

Symmetry codes: (i) 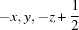; (ii) 
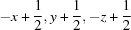
.

**Fig. 1.**
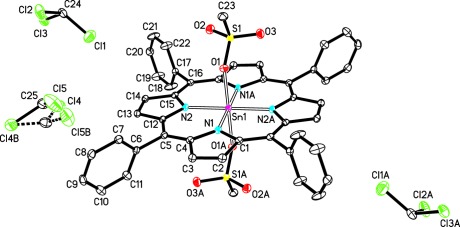
The molecular structure of [Sn(C44H28N4)(CH3SO3)2].3CHCl3 with the atom numbering scheme. Displacement ellipsoids are drawn at the 50% probability level. H atoms are omitted for clarity. [Symmetry codes: (A) -x, -y, -z; (B) -x, y, -z + 0.5.]

**Fig. 2.**
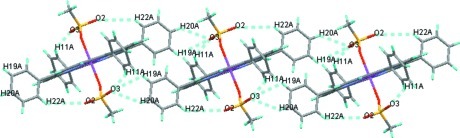
View of the one-dimensional chain along the b-axis. Hydrogen-bonding interactions are drawn with dashed lines.

**Fig. 3.**
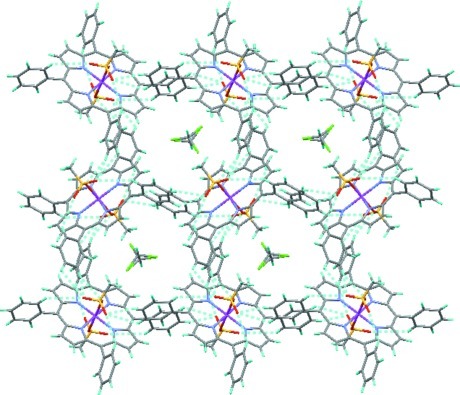
View along the a-axis of the two-dimensional structure. Hydrogen-bond interactions are drawn with dashed lines.

**Fig. 4.**
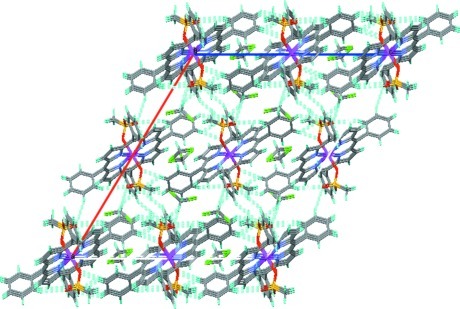
View along b-axis of the three-dimensional structure. Hydrogen-bond interactions are drawn with dashed lines.

## supplementary crystallographic information

### Comment

In tin(IV) porphyrin chemistry, a number of compounds have been synthesized through variation of the axial ligands such as hydroxide, alkoxides, halides, nitrate, perchlorate or carboxylates (Arnold & Blok, 2004). Among these compounds, hydroxido-tin(IV) porphyrins have been employed as useful precursors for the preparation of various tin(IV) prophyrin complexes bearing oxygen donor ligands preferentially (Kim *et al.*, 2004, 2005, 2007, 2009). In this order, we have studied the reactivity behaviour of hydroxido-tin(IV) porphyrins with various acidic compounds including sulfonic acid derivatives. Here we report the synthesis, characterization and X-ray structural study of the title solvate [Sn(C_44_H_28_N_4_)(CH_3_SO_3_)_2_]^.^3CHCl_3_ or [Sn(TPP)(CH_3_SO_3_)_2_]^.^3CHCl_3_ (TPP = tetraphenylporphyrinato dianion).

The characterization of the title compound has been carried out with ^1^H NMR spectroscopy (see supplementary materials) as well as with X-ray crystal structure analysis. The molecular structure of Sn(TPP)(CH_3_SO_3_)_2_ shown in Figure 1 exhibits an octahedral geometry around the tin(IV) ion (site symmetry 1). The equatorial plane is formed by four N atoms of the porphyrin ring while the axial positions are occupied by methanesulfonate groups. The methanesulfonate groups are unidentately coordinating to the tin atom. The bond lengths, Sn1—N1 = 2.074 (2), Sn1—N2 = 2.081 (2) Å and angles, N1—Sn1—N2 = 89.99 (8)°, N1—Sn1—N2^i^ = 90.01 (8)°, N1—Sn1—N1^i^ = 180.00 (7)° and O1—Sn1—O1^i^ = 180.0°, associated with these atoms well corroborate the fact related to an octahedral coordination environment around the tin atom. The bond length Sn—*O_methanesulfonato_* (2.1184 (18) Å) is significantly longer than Sn—*O_acetato_* (2.086 (5) Å) in Sn(TPP)(CH_3_CO_2_)_2_ (Liu *et al.*, 1996), which reflects a less basic character of the methanesulfonate group than of the acetate group. On comparison with the reported Sn(TPP) complex bearing CF_3_SO_3_^-^ groups (Smith *et al.*, 1991), this comound crystallizes as a diaqua-complex where the two water molecules are coordinating to the tin atom at the axial sites while the two CF_3_SO_3_^-^ groups act as counter anions. The comparative study of these two anionic sulfonate groups evidently reveals that the methanesulfonate anion has a more basic character than the trifluoromethanesulfonate anion.

In the crystal structure of [Sn(C_44_H_28_N_4_)(CH_3_SO_3_)_2_]^.^3CHCl_3_ the presence of intramolecular and intermolecular hydrogen bonding (which lies in the range of moderate to weak bonding) is evident. The molecular structure shows the presence of one intramolecular hydrogen bond (C11—H11A···O3 = 2.846 Å) between the phenyl H11A atom and the O3 atom of the methanesulfonato ligand. Two additional hydrogen bonds (C20—H20A···O3 = 2.626, C19—H19A···O3 = 3.012 Å) involving H19A, H20A atoms of the phenyl groups and O3 atoms of the methanesulfonato ligands form a chain structure along the *b*-axis (Figure 2). Further, these adjacent one-dimensional chains interact with each other *via* additional hydrogen bonds, resulting in two-dimensional (C8—H8A···O2 = 2.552, C8—H8A···O1 = 2.769 Å) (Figure 3) and three-dimensional (C10—H10A···O3 = 3.065 Å) (Figure 4) structural motifs. The view of three-dimensional supramolecular assembly along the *b*-axis (in *ac*-plane) displays the presence of infinite channels which are filled by chloroform molecules. The asymmetric unit is associated with two chloroform molecules present in the three-dimensional channels as free solvent molecules. Another chloroform molecules in the asymmetric unit is present in a weakly hydrogen-bonded state (C24—H24A···O3 = 2.720, C24—H24A···O2 = 2.352 Å) with the oxygen atoms of the methanesulfonato ligands of adjacent asymmetric units.

### Experimental

A mixture of Sn(TPP)(OH)_2_ (0.150 g, 1.96 × 10 ^-4^ mol) and methanesulfonic acid (0.0376 g, 3.92 × 10 ^-4^ mol) in dry chloroform (20 ml) was stirred at room temperature for 8 h. The solvent was removed and the crude material was stirred in dry hexane for 4 h. The precipitated compound was filtered and dried *in vacuo*. The product was further recrystallized by slow diffusion of *n*-hexane into a chloroform solution of the compound. Yield: 75%. Mp > 300° C. ^1^H NMR (400 MHz, CDCl_3_, SiMe_4_): *δ* -0.50 (6*H*, s, CH_3_), 7.79–7.86 (12*H*, Ar—H), 8.29 (8*H*, d, ^3^*J*_H—H_ = 6.56 Hz, Ar—H), 9.17 (8*H*, s, ^4^*J*_H—Sn_ = 14.8 Hz, *β*-pyrrolic H).

### Refinement

The occupancy of chlorine atom has been distributed at two atomic sites in the ratios of 50:50 with total site occupancy of 1.00. All non-hydrogen atoms were refined anisotropically, and hydrogen atoms were added to their geometrically ideal positions.

### Crystal data

**Table:**

[Sn(C_44_H_28_N_4_)(CH_3_O_3_S)_2_]·3CHCl_3_	*F*(000) = 2568
*M**_r_* = 1279.69	*D*_x_ = 1.610 Mg m^−^^3^
Monoclinic, *C*2/*c*	Mo *K*α radiation, λ = 0.71073 Å
Hall symbol: -C 2yc	Cell parameters from 9494 reflections
*a* = 25.379 (2) Å	θ = 2.5–27.6°
*b* = 11.6269 (9) Å	µ = 1.07 mm^−^^1^
*c* = 20.860 (3) Å	*T* = 150 K
β = 120.934 (1)°	Block, violet
*V* = 5279.9 (9) Å^3^	0.26 × 0.19 × 0.16 mm
*Z* = 4	

### Data collection

**Table:**

Bruker APEXII CCD diffractometer	5192 independent reflections
Radiation source: Turbo X-ray	4525 reflections with *I* > 2σ(*I*)
Multilayer monochromator	*R*_int_ = 0.035
φ and ω scans	θ_max_ = 26.0°, θ_min_ = 1.9°
Absorption correction: multi-scan (*SADABS*; Bruker, 2009)	*h* = −31→31
*T*_min_ = 0.765, *T*_max_ = 0.848	*k* = −14→14
22613 measured reflections	*l* = −24→25

### Refinement

**Table:**

Refinement on *F*^2^	Primary atom site location: structure-invariant direct methods
Least-squares matrix: full	Secondary atom site location: difference Fourier map
*R*[*F*^2^ > 2σ(*F*^2^)] = 0.033	Hydrogen site location: inferred from neighbouring sites
*wR*(*F*^2^) = 0.088	H-atom parameters constrained
*S* = 1.04	*w* = 1/[σ^2^(*F*_o_^2^) + (0.0404*P*)^2^ + 12.8121*P*] where *P* = (*F*_o_^2^ + 2*F*_c_^2^)/3
5192 reflections	(Δ/σ)_max_ < 0.001
331 parameters	Δρ_max_ = 0.91 e Å^−^^3^
0 restraints	Δρ_min_ = −0.86 e Å^−^^3^

### Special details

**Table:**

Geometry. All e.s.d.'s (except the e.s.d. in the dihedral angle between two l.s. planes) are estimated using the full covariance matrix. The cell e.s.d.'s are taken into account individually in the estimation of e.s.d.'s in distances, angles and torsion angles; correlations between e.s.d.'s in cell parameters are only used when they are defined by crystal symmetry. An approximate (isotropic) treatment of cell e.s.d.'s is used for estimating e.s.d.'s involving l.s. planes.
Refinement. Refinement of *F*^2^ against ALL reflections. The weighted *R*-factor *wR* and goodness of fit *S* are based on *F*^2^, conventional *R*-factors *R* are based on *F*, with *F* set to zero for negative *F*^2^. The threshold expression of *F*^2^ > 2σ(*F*^2^) is used only for calculating *R*-factors(gt) *etc*. and is not relevant to the choice of reflections for refinement. *R*-factors based on *F*^2^ are statistically about twice as large as those based on *F*, and *R*– factors based on ALL data will be even larger.

### Fractional atomic coordinates and isotropic or equivalent isotropic displacement parameters (Å^2^)

**Table:**

	*x*	*y*	*z*	*U*_iso_*/*U*_eq_	Occ. (<1)
Sn1	0.0000	0.0000	0.0000	0.02057 (8)	
S1	0.14343 (3)	−0.07734 (6)	0.12355 (4)	0.02780 (15)	
O1	0.07563 (8)	−0.07458 (16)	0.09584 (10)	0.0297 (4)	
O2	0.16993 (10)	0.03546 (19)	0.14721 (12)	0.0408 (5)	
O3	0.15589 (10)	−0.13277 (19)	0.07136 (12)	0.0416 (5)	
N1	−0.05688 (9)	−0.08286 (17)	0.02948 (11)	0.0233 (4)	
N2	0.00374 (9)	0.14274 (17)	0.06239 (11)	0.0228 (4)	
C1	−0.08282 (12)	−0.1898 (2)	0.00474 (14)	0.0256 (5)	
C2	−0.12325 (13)	−0.2100 (2)	0.03274 (15)	0.0299 (6)	
H2A	−0.1465	−0.2779	0.0255	0.036*	
C3	−0.12243 (12)	−0.1162 (2)	0.07092 (15)	0.0291 (6)	
H3A	−0.1456	−0.1058	0.0946	0.035*	
C4	−0.08066 (12)	−0.0349 (2)	0.06968 (14)	0.0250 (5)	
C5	−0.06792 (12)	0.0747 (2)	0.10157 (14)	0.0247 (5)	
C6	−0.10283 (12)	0.1116 (2)	0.13798 (14)	0.0261 (5)	
C7	−0.08757 (13)	0.0719 (3)	0.20824 (15)	0.0335 (6)	
H7A	−0.0543	0.0199	0.2342	0.040*	
C8	−0.12063 (14)	0.1076 (3)	0.24057 (17)	0.0408 (7)	
H8A	−0.1093	0.0815	0.2892	0.049*	
C9	−0.16933 (15)	0.1800 (3)	0.20320 (19)	0.0429 (8)	
H9A	−0.1922	0.2031	0.2254	0.051*	
C10	−0.18547 (16)	0.2199 (3)	0.1334 (2)	0.0464 (8)	
H10A	−0.2194	0.2706	0.1076	0.056*	
C11	−0.15213 (14)	0.1861 (3)	0.10063 (17)	0.0372 (7)	
H11A	−0.1632	0.2141	0.0525	0.045*	
C12	−0.02762 (11)	0.1554 (2)	0.09967 (13)	0.0239 (5)	
C13	−0.01197 (12)	0.2649 (2)	0.13580 (15)	0.0279 (6)	
H13A	−0.0268	0.2958	0.1657	0.033*	
C14	0.02779 (12)	0.3169 (2)	0.12005 (15)	0.0271 (5)	
H14A	0.0460	0.3904	0.1372	0.033*	
C15	0.03759 (11)	0.2412 (2)	0.07293 (14)	0.0239 (5)	
C16	0.07386 (11)	0.2634 (2)	0.04160 (14)	0.0243 (5)	
C17	0.10721 (12)	0.3760 (2)	0.06105 (14)	0.0248 (5)	
C18	0.08175 (16)	0.4711 (3)	0.0177 (2)	0.0612 (12)	
H18A	0.0417	0.4664	−0.0253	0.073*	
C19	0.11340 (17)	0.5748 (3)	0.0354 (2)	0.0643 (12)	
H19A	0.0947	0.6405	0.0048	0.077*	
C20	0.17068 (14)	0.5828 (2)	0.09582 (18)	0.0375 (7)	
H20A	0.1926	0.6534	0.1077	0.045*	
C21	0.19643 (16)	0.4888 (3)	0.1391 (2)	0.0595 (11)	
H21A	0.2366	0.4940	0.1819	0.071*	
C22	0.16519 (15)	0.3853 (3)	0.1222 (2)	0.0525 (9)	
H22A	0.1841	0.3202	0.1531	0.063*	
C23	0.17079 (15)	−0.1659 (3)	0.20286 (17)	0.0421 (7)	
H23A	0.2154	−0.1752	0.2265	0.063*	
H23B	0.1509	−0.2414	0.1878	0.063*	
H23C	0.1613	−0.1304	0.2384	0.063*	
C24	0.22194 (17)	0.5661 (3)	0.3870 (2)	0.0504 (8)	
H24A	0.2647	0.5432	0.4016	0.061*	
Cl1	0.17224 (7)	0.51173 (11)	0.29855 (8)	0.0916 (4)	
Cl2	0.20717 (7)	0.51221 (9)	0.45494 (8)	0.0788 (4)	
Cl3	0.21860 (5)	0.71697 (8)	0.38628 (6)	0.0621 (3)	
C25	0.0212 (4)	0.6766 (7)	0.2678 (6)	0.070 (2)	0.50
H25	0.0622	0.6907	0.3138	0.084*	0.50
Cl4	0.02877 (7)	0.75684 (14)	0.20677 (9)	0.1048 (5)	
Cl5	0.0137 (3)	0.5484 (3)	0.2778 (3)	0.146 (3)	0.50

### Atomic displacement parameters (Å^2^)

**Table:**

	*U*^11^	*U*^22^	*U*^33^	*U*^12^	*U*^13^	*U*^23^
Sn1	0.02249 (13)	0.01973 (13)	0.02126 (13)	−0.00515 (9)	0.01251 (10)	−0.00170 (9)
S1	0.0274 (3)	0.0302 (3)	0.0260 (3)	0.0002 (3)	0.0140 (3)	0.0005 (3)
O1	0.0265 (9)	0.0327 (10)	0.0275 (10)	−0.0020 (8)	0.0122 (8)	0.0053 (8)
O2	0.0397 (12)	0.0418 (11)	0.0446 (12)	−0.0139 (10)	0.0244 (10)	−0.0095 (10)
O3	0.0509 (13)	0.0439 (12)	0.0353 (11)	0.0098 (10)	0.0259 (10)	0.0010 (9)
N1	0.0262 (11)	0.0218 (10)	0.0237 (11)	−0.0053 (8)	0.0140 (9)	−0.0017 (8)
N2	0.0259 (11)	0.0218 (10)	0.0241 (11)	−0.0060 (8)	0.0152 (9)	−0.0030 (8)
C1	0.0266 (13)	0.0231 (12)	0.0258 (13)	−0.0050 (10)	0.0126 (11)	0.0008 (10)
C2	0.0332 (14)	0.0280 (13)	0.0348 (15)	−0.0083 (11)	0.0220 (12)	−0.0005 (11)
C3	0.0315 (14)	0.0304 (14)	0.0322 (14)	−0.0073 (11)	0.0213 (12)	−0.0028 (11)
C4	0.0256 (13)	0.0277 (12)	0.0233 (13)	−0.0044 (10)	0.0137 (11)	0.0011 (10)
C5	0.0261 (13)	0.0280 (13)	0.0212 (12)	−0.0025 (10)	0.0131 (11)	0.0002 (10)
C6	0.0280 (13)	0.0268 (13)	0.0277 (13)	−0.0093 (10)	0.0173 (11)	−0.0062 (10)
C7	0.0316 (14)	0.0431 (16)	0.0285 (14)	−0.0044 (12)	0.0174 (12)	0.0006 (12)
C8	0.0428 (17)	0.0565 (19)	0.0331 (16)	−0.0188 (15)	0.0267 (14)	−0.0111 (14)
C9	0.0468 (18)	0.0452 (18)	0.056 (2)	−0.0129 (15)	0.0406 (17)	−0.0177 (15)
C10	0.0442 (18)	0.0385 (17)	0.068 (2)	0.0040 (14)	0.0366 (18)	0.0015 (16)
C11	0.0398 (16)	0.0370 (16)	0.0408 (17)	0.0028 (13)	0.0250 (14)	0.0057 (13)
C12	0.0270 (13)	0.0249 (12)	0.0202 (12)	−0.0012 (10)	0.0124 (10)	0.0002 (10)
C13	0.0297 (13)	0.0272 (13)	0.0290 (14)	−0.0026 (11)	0.0167 (12)	−0.0045 (11)
C14	0.0305 (13)	0.0211 (12)	0.0286 (13)	−0.0039 (10)	0.0143 (11)	−0.0027 (10)
C15	0.0235 (12)	0.0222 (12)	0.0230 (12)	−0.0034 (10)	0.0098 (10)	−0.0005 (10)
C16	0.0247 (12)	0.0220 (12)	0.0249 (13)	−0.0036 (10)	0.0119 (11)	0.0006 (10)
C17	0.0272 (13)	0.0219 (12)	0.0293 (13)	−0.0052 (10)	0.0174 (11)	−0.0025 (10)
C18	0.0387 (18)	0.0378 (18)	0.063 (2)	−0.0121 (15)	−0.0052 (17)	0.0162 (17)
C19	0.049 (2)	0.0291 (16)	0.074 (3)	−0.0094 (15)	0.0023 (19)	0.0189 (17)
C20	0.0394 (16)	0.0238 (13)	0.0507 (18)	−0.0099 (12)	0.0242 (15)	−0.0067 (13)
C21	0.0374 (18)	0.0354 (18)	0.063 (2)	−0.0132 (14)	−0.0052 (17)	0.0039 (16)
C22	0.0418 (18)	0.0301 (16)	0.052 (2)	−0.0078 (13)	0.0004 (16)	0.0109 (14)
C23	0.0398 (17)	0.0498 (18)	0.0309 (15)	0.0100 (14)	0.0140 (13)	0.0115 (14)
C24	0.056 (2)	0.053 (2)	0.055 (2)	0.0161 (17)	0.0373 (18)	0.0061 (17)
Cl1	0.0975 (10)	0.0843 (9)	0.0691 (8)	−0.0046 (7)	0.0257 (7)	−0.0116 (6)
Cl2	0.1253 (11)	0.0547 (6)	0.0981 (9)	0.0140 (6)	0.0872 (9)	0.0121 (5)
Cl3	0.0740 (6)	0.0512 (5)	0.0733 (6)	0.0138 (5)	0.0466 (6)	0.0105 (5)
C25	0.059 (5)	0.060 (5)	0.083 (7)	−0.006 (3)	0.031 (5)	−0.009 (4)
Cl4	0.0796 (9)	0.1124 (11)	0.1096 (11)	0.0073 (8)	0.0395 (8)	0.0276 (9)
Cl5	0.169 (6)	0.0757 (16)	0.248 (8)	0.053 (3)	0.146 (6)	0.069 (3)

### Geometric parameters (Å, º)

**Table:**

Sn1—N1	2.074 (2)	C12—C13	1.427 (4)
Sn1—N1^i^	2.074 (2)	C13—C14	1.354 (4)
Sn1—N2^i^	2.081 (2)	C13—H13A	0.9500
Sn1—N2	2.081 (2)	C14—C15	1.433 (4)
Sn1—O1	2.1184 (18)	C14—H14A	0.9500
Sn1—O1^i^	2.1185 (18)	C15—C16	1.400 (4)
S1—O3	1.434 (2)	C16—C1^i^	1.394 (4)
S1—O2	1.440 (2)	C16—C17	1.498 (3)
S1—O1	1.5077 (19)	C17—C18	1.363 (4)
S1—C23	1.760 (3)	C17—C22	1.369 (4)
N1—C1	1.377 (3)	C18—C19	1.389 (5)
N1—C4	1.378 (3)	C18—H18A	0.9500
N2—C12	1.377 (3)	C19—C20	1.352 (5)
N2—C15	1.380 (3)	C19—H19A	0.9500
C1—C16^i^	1.394 (4)	C20—C21	1.353 (4)
C1—C2	1.437 (4)	C20—H20A	0.9500
C2—C3	1.344 (4)	C21—C22	1.383 (4)
C2—H2A	0.9500	C21—H21A	0.9500
C3—C4	1.431 (3)	C22—H22A	0.9500
C3—H3A	0.9500	C23—H23A	0.9800
C4—C5	1.397 (4)	C23—H23B	0.9800
C5—C12	1.403 (3)	C23—H23C	0.9800
C5—C6	1.496 (4)	C24—Cl1	1.733 (4)
C6—C11	1.386 (4)	C24—Cl2	1.756 (4)
C6—C7	1.388 (4)	C24—Cl3	1.756 (4)
C7—C8	1.383 (4)	C24—H24A	1.0000
C7—H7A	0.9500	C25—Cl5	1.530 (9)
C8—C9	1.361 (5)	C25—Cl4	1.667 (10)
C8—H8A	0.9500	C25—Cl4^ii^	1.858 (9)
C9—C10	1.374 (5)	C25—H25	1.0000
C9—H9A	0.9500	Cl4—C25^ii^	1.858 (9)
C10—C11	1.390 (4)	Cl5—Cl5^ii^	0.999 (10)
C10—H10A	0.9500	Cl5—C25^ii^	1.745 (9)
C11—H11A	0.9500		
			
N1—Sn1—N1^i^	180.00 (7)	C6—C11—C10	120.1 (3)
N1—Sn1—N2^i^	90.01 (8)	C6—C11—H11A	119.9
N1^i^—Sn1—N2^i^	89.99 (8)	C10—C11—H11A	119.9
N1—Sn1—N2	89.99 (8)	N2—C12—C5	125.8 (2)
N1^i^—Sn1—N2	90.01 (8)	N2—C12—C13	108.0 (2)
N2^i^—Sn1—N2	180.0	C5—C12—C13	126.2 (2)
N1—Sn1—O1	87.77 (8)	C14—C13—C12	108.1 (2)
N1^i^—Sn1—O1	92.23 (8)	C14—C13—H13A	126.0
N2^i^—Sn1—O1	89.52 (8)	C12—C13—H13A	126.0
N2—Sn1—O1	90.48 (8)	C13—C14—C15	107.8 (2)
N1—Sn1—O1^i^	92.23 (8)	C13—C14—H14A	126.1
N1^i^—Sn1—O1^i^	87.77 (8)	C15—C14—H14A	126.1
N2^i^—Sn1—O1^i^	90.47 (8)	N2—C15—C16	125.7 (2)
N2—Sn1—O1^i^	89.53 (8)	N2—C15—C14	107.8 (2)
O1—Sn1—O1^i^	180.0	C16—C15—C14	126.5 (2)
O3—S1—O2	115.02 (13)	C1^i^—C16—C15	126.3 (2)
O3—S1—O1	111.86 (12)	C1^i^—C16—C17	117.0 (2)
O2—S1—O1	110.60 (12)	C15—C16—C17	116.7 (2)
O3—S1—C23	108.45 (14)	C18—C17—C22	118.0 (3)
O2—S1—C23	108.75 (15)	C18—C17—C16	121.4 (2)
O1—S1—C23	101.19 (13)	C22—C17—C16	120.6 (2)
S1—O1—Sn1	132.25 (11)	C17—C18—C19	121.0 (3)
C1—N1—C4	108.6 (2)	C17—C18—H18A	119.5
C1—N1—Sn1	125.42 (17)	C19—C18—H18A	119.5
C4—N1—Sn1	125.57 (16)	C20—C19—C18	120.3 (3)
C12—N2—C15	108.3 (2)	C20—C19—H19A	119.8
C12—N2—Sn1	125.80 (16)	C18—C19—H19A	119.8
C15—N2—Sn1	125.87 (16)	C19—C20—C21	119.1 (3)
N1—C1—C16^i^	126.7 (2)	C19—C20—H20A	120.4
N1—C1—C2	107.4 (2)	C21—C20—H20A	120.4
C16^i^—C1—C2	125.9 (2)	C20—C21—C22	121.0 (3)
C3—C2—C1	108.1 (2)	C20—C21—H21A	119.5
C3—C2—H2A	125.9	C22—C21—H21A	119.5
C1—C2—H2A	125.9	C17—C22—C21	120.5 (3)
C2—C3—C4	108.2 (2)	C17—C22—H22A	119.8
C2—C3—H3A	125.9	C21—C22—H22A	119.8
C4—C3—H3A	125.9	S1—C23—H23A	109.5
N1—C4—C5	126.3 (2)	S1—C23—H23B	109.5
N1—C4—C3	107.7 (2)	H23A—C23—H23B	109.5
C5—C4—C3	125.9 (2)	S1—C23—H23C	109.5
C4—C5—C12	126.2 (2)	H23A—C23—H23C	109.5
C4—C5—C6	116.8 (2)	H23B—C23—H23C	109.5
C12—C5—C6	117.0 (2)	Cl1—C24—Cl2	112.9 (2)
C11—C6—C7	118.9 (3)	Cl1—C24—Cl3	110.4 (2)
C11—C6—C5	119.5 (2)	Cl2—C24—Cl3	109.52 (19)
C7—C6—C5	121.6 (2)	Cl1—C24—H24A	107.9
C8—C7—C6	120.3 (3)	Cl2—C24—H24A	107.9
C8—C7—H7A	119.8	Cl3—C24—H24A	107.9
C6—C7—H7A	119.8	Cl5—C25—Cl4	136.0 (7)
C9—C8—C7	120.4 (3)	Cl5—C25—Cl4^ii^	107.4 (6)
C9—C8—H8A	119.8	Cl4—C25—Cl4^ii^	107.5 (4)
C7—C8—H8A	119.8	Cl5—C25—H25	99.8
C8—C9—C10	120.2 (3)	Cl4—C25—H25	99.8
C8—C9—H9A	119.9	Cl4^ii^—C25—H25	99.8
C10—C9—H9A	119.9	C25—Cl4—C25^ii^	30.5 (5)
C9—C10—C11	120.0 (3)	Cl5^ii^—Cl5—C25	84.5 (4)
C9—C10—H10A	120.0	Cl5^ii^—Cl5—C25^ii^	60.8 (4)
C11—C10—H10A	120.0	C25—Cl5—C25^ii^	32.7 (6)
			
O3—S1—O1—Sn1	−60.68 (19)	C6—C7—C8—C9	1.5 (4)
O2—S1—O1—Sn1	68.94 (18)	C7—C8—C9—C10	−1.2 (5)
C23—S1—O1—Sn1	−175.95 (16)	C8—C9—C10—C11	0.2 (5)
N1—Sn1—O1—S1	172.62 (16)	C7—C6—C11—C10	0.0 (4)
N1^i^—Sn1—O1—S1	−7.38 (16)	C5—C6—C11—C10	179.2 (3)
N2^i^—Sn1—O1—S1	82.59 (16)	C9—C10—C11—C6	0.4 (5)
N2—Sn1—O1—S1	−97.41 (16)	C15—N2—C12—C5	178.9 (2)
N2^i^—Sn1—N1—C1	−2.8 (2)	Sn1—N2—C12—C5	−0.5 (4)
N2—Sn1—N1—C1	177.2 (2)	C15—N2—C12—C13	−1.0 (3)
O1—Sn1—N1—C1	−92.3 (2)	Sn1—N2—C12—C13	179.53 (17)
O1^i^—Sn1—N1—C1	87.7 (2)	C4—C5—C12—N2	3.9 (4)
N2^i^—Sn1—N1—C4	−174.7 (2)	C6—C5—C12—N2	−173.0 (2)
N2—Sn1—N1—C4	5.3 (2)	C4—C5—C12—C13	−176.2 (3)
O1—Sn1—N1—C4	95.8 (2)	C6—C5—C12—C13	6.9 (4)
O1^i^—Sn1—N1—C4	−84.2 (2)	N2—C12—C13—C14	0.3 (3)
N1—Sn1—N2—C12	−3.1 (2)	C5—C12—C13—C14	−179.6 (3)
N1^i^—Sn1—N2—C12	176.9 (2)	C12—C13—C14—C15	0.5 (3)
O1—Sn1—N2—C12	−90.9 (2)	C12—N2—C15—C16	−177.5 (2)
O1^i^—Sn1—N2—C12	89.1 (2)	Sn1—N2—C15—C16	2.0 (4)
N1—Sn1—N2—C15	177.5 (2)	C12—N2—C15—C14	1.3 (3)
N1^i^—Sn1—N2—C15	−2.5 (2)	Sn1—N2—C15—C14	−179.23 (16)
O1—Sn1—N2—C15	89.8 (2)	C13—C14—C15—N2	−1.1 (3)
O1^i^—Sn1—N2—C15	−90.2 (2)	C13—C14—C15—C16	177.6 (3)
C4—N1—C1—C16^i^	175.8 (3)	N2—C15—C16—C1^i^	−0.9 (4)
Sn1—N1—C1—C16^i^	2.8 (4)	C14—C15—C16—C1^i^	−179.4 (3)
C4—N1—C1—C2	−1.6 (3)	N2—C15—C16—C17	−179.5 (2)
Sn1—N1—C1—C2	−174.65 (17)	C14—C15—C16—C17	1.9 (4)
N1—C1—C2—C3	1.8 (3)	C1^i^—C16—C17—C18	89.4 (4)
C16^i^—C1—C2—C3	−175.7 (3)	C15—C16—C17—C18	−91.8 (4)
C1—C2—C3—C4	−1.2 (3)	C1^i^—C16—C17—C22	−88.7 (4)
C1—N1—C4—C5	−177.3 (2)	C15—C16—C17—C22	90.1 (3)
Sn1—N1—C4—C5	−4.2 (4)	C22—C17—C18—C19	−0.7 (6)
C1—N1—C4—C3	0.9 (3)	C16—C17—C18—C19	−178.9 (4)
Sn1—N1—C4—C3	173.93 (17)	C17—C18—C19—C20	0.8 (7)
C2—C3—C4—N1	0.2 (3)	C18—C19—C20—C21	−0.6 (7)
C2—C3—C4—C5	178.4 (3)	C19—C20—C21—C22	0.3 (7)
N1—C4—C5—C12	−1.2 (4)	C18—C17—C22—C21	0.4 (6)
C3—C4—C5—C12	−179.1 (3)	C16—C17—C22—C21	178.6 (4)
N1—C4—C5—C6	175.6 (2)	C20—C21—C22—C17	−0.2 (7)
C3—C4—C5—C6	−2.2 (4)	Cl5—C25—Cl4—C25^ii^	80.4 (10)
C4—C5—C6—C11	−101.8 (3)	Cl4^ii^—C25—Cl4—C25^ii^	−60.6 (7)
C12—C5—C6—C11	75.3 (3)	Cl4—C25—Cl5—Cl5^ii^	−39.6 (13)
C4—C5—C6—C7	77.4 (3)	Cl4^ii^—C25—Cl5—Cl5^ii^	101.5 (9)
C12—C5—C6—C7	−105.5 (3)	Cl4—C25—Cl5—C25^ii^	−80.3 (14)
C11—C6—C7—C8	−0.9 (4)	Cl4^ii^—C25—Cl5—C25^ii^	60.8 (10)
C5—C6—C7—C8	179.9 (3)		

Symmetry codes: (i) −*x*, −*y*, −*z*; (ii) −*x*, *y*, −*z*+1/2.

### Hydrogen-bond geometry (Å, º)

**Table:**

*D*—H···*A*	*D*—H	H···*A*	*D*···*A*	*D*—H···*A*
C8—H8*A*···O2^ii^	0.95	2.55	3.280 (4)	134
C24—H24*A*···O2^iii^	1.00	2.35	3.191 (4)	141

Symmetry codes: (ii) −*x*, *y*, −*z*+1/2; (iii) −*x*+1/2, *y*+1/2, −*z*+1/2.
